# Acute Toxoplasmosis among Canadian Deer Hunters Associated with Consumption of Undercooked Deer Meat Hunted in the United States

**DOI:** 10.3201/eid2602.191218

**Published:** 2020-02

**Authors:** Colette Gaulin, Danielle Ramsay, Karine Thivierge, Joanne Tataryn, Ariane Courville, Catherine Martin, Patricia Cunningham, Joane Désilets, Diane Morin, Réjean Dion

**Affiliations:** Ministère de la Santé et des Services Sociaux, Quebec City, Quebec, Canada (C. Gaulin);; Ministère de l’Agriculture, des Pêcheries, et de l’Alimentation du Quebec, Quebec City (D. Ramsay);; Institut National de Santé Publique du Québec, Sainte-Anne-de-Bellevue, Quebec, Canada (K. Thivierge, R. Dion);; Canadian Public Health Agency, Ottawa, Ontario, Canada (J. Tataryn);; Direction de Santé Publique Gaspésie–Îles-de-la-Madeleine, Gaspé, Quebec, Canada (A. Courville);; Direction de la Santé Publique du Bas St-Laurent, Quebec City (C. Martin);; Direction de Santé Publique Lanaudière, Joliette, Quebec, Canada (P. Cunningham, J. Désilets);; Centre Intégré de Santé et de Services Sociaux de Chaudière-Appalaches, Sainte-Marie de Beauce, Quebec, Canada (D. Morin)

**Keywords:** toxoplasmosis, outbreak, Toxoplasma gondii, parasites, epidemiology, infection, foodborne diseases, food safety, deer hunters, undercooked deer meat, zoonoses, Quebec, Canada, Illinois, United States

## Abstract

We conducted a recent investigation in Quebec, Canada, concerning Canadian deer hunters who went to the United States to hunt deer and returned with symptoms of fever, severe headache, myalgia, and articular pain of undetermined etiology. Further investigation identified that a group of 10 hunters from Quebec attended a hunting retreat in Illinois (USA) during November 22–December 4, 2018. Six of the 10 hunters had similar symptoms and illness onset dates. Serologic tests indicated a recent toxoplasmosis infection for all symptomatic hunters, and the risk factor identified was consumption of undercooked deer meat. Among asymptomatic hunters, 2 were already immune to toxoplasmosis, 1 was not immune, and the immune status of 1 remains unknown. Outbreaks of acute toxoplasmosis infection are rare in North America, but physicians should be aware that such outbreaks could become more common.

## Abstract 2

Health professionals should be aware that such outbreaks might be more common in the future

*Toxoplasma gondii* is one of the most common zoonotic parasites and can cause serious illness in humans and other animals worldwide (*1**–**3*). It can infect virtually all warm-blooded animals, including birds, livestock, marine mammals, and humans (*2*). Felids are the definitive hosts of *T. gondii*, meaning they are the only animals in which replication can result in the production of oocysts (eggs), which are then shed in the feces (*2**,**3*). Felids are essential to the epidemiology of this parasite (*2*).

Human *T. gondii* infection is caused by ingestion of tissue cysts in undercooked meat; ingestion of soil, water, or food contaminated with oocysts; or, less frequently, directly from feline feces (*3**–**8*). Frequency of human infection might vary substantially by region because of ecologic, social, and cultural factors (*3*).

*T. gondii* infection acquired after birth can be asymptomatic in humans. Symptoms appear mostly in immunocompromised persons. When symptoms develop, they are nonspecific and include malaise, fever, headache, sore throat, arthralgia, and myalgia.

Lymphadenopathy, frequently cervical, is the most common sign (*3*). Persons remain infected for life (*3*). Reactivation of the disease is possible sometimes years later. However, outbreaks of acute toxoplasmosis seem to be rare.

Cervids can be infected by *T. gondii* (*2**,**7**,**9**–**12*). Cases of clinical toxoplasmosis have been documented in humans who had consumed undercooked venison (*13*). Toxoplasmosis infection was documented in 1 Alabama and 2 South Carolina deer hunters during 1980 (*14*). A recent outbreak was reported in the United States during 2017 (*15*). However, it is quite rare to observe a cluster of cases related to undercooked deer meat, particularly related to consumption of venison. In addition, cysts and oocysts of *T. gondii* are destroyed by freezing. We report an acute toxoplasmosis outbreak in Quebec, Canada, associated with consumption of venison. We conducted an investigation to determine the outbreak magnitude, describe illness-related factors, and coordinate *Toxoplasma* spp. diagnostic testing.

## Background

On December 20, 2018, public health authorities in Quebec were alerted regarding a patient with fever, severe headache, myalgia, and articular pain of undetermined etiology. The first symptom onset occurred on December 8. The patient required hospitalization; medical history showed no chronic or immunologic disease.

Further investigation identified that this patient and 9 hunter companions from Quebec attended a hunting retreat in Illinois (USA) during November 22–December 4, ending the week before illness began. Six of the 10 hunters had similar symptoms and illness onset dates. Case-patients reported consuming undercooked venison during the retreat. Hunters were tested for Q fever, hepatitis E, leptospirosis, brucellosis, Lyme disease, and toxoplasmosis. Serologic tests indicated recent toxoplasmosis infections.

## Material and Methods

### Case Definition

A confirmed case was defined by serologic test results (IgM positive for toxoplasmosis and a low-avidity test result). These results were consistent with a recently acquired *Toxoplasma* spp. infection in a person who had clinical symptoms compatible with toxoplasmosis after attending the deer hunting retreat during November 22–December 4, 2018.

### Epidemiologic Investigation

On December 20, 2018, the Direction de la Vigie Sanitaire at the Ministère de la Santé et des Services Sociaux (Ministry of Health in Quebec) initiated an investigation. This investigation was conducted in collaboration with the Ministère de l’Agriculture, des Pêcheries et de l’Alimentation du Quebec (MAPAQ: Ministry of Agriculture, Fisheries, and Food of Quebec), public health units, and the Laboratoire de Santé Publique du Quebec (LSPQ: Public Health Laboratory in Quebec).

All 10 hunting companions who attended the retreat in Illinois were interviewed. The following information was collected from each attendee, symptomatic or asymptomatic: demographic information; description of activities at the outfitter, including deer hunting and evisceration; food consumed on site, including deer meat and how it was eaten (raw, undercooked, or well done); consumption of water; and possible exposure to ticks or other animals. For persons who had symptoms, we obtained information on onset dates and symptoms. Attendees were interviewed mostly by public health nurses or medical microbiologists and infectious disease physicians.

### Food Inspection Services

Deer meat harvested during the trip was available, and we collected specimens from hunter households. Meat samples were collected by the food inspection services at the MAPAQ and analyzed by the Molecular Diagnosis Laboratory at the Veterinary School at the University of Montreal (Montreal, Quebec, Canada) by using standardized and adapted methods (*16*).

### Serologic Tests

We tested symptomatic and asymptomatic hunters by using serologic analysis for toxoplasmosis, brucellosis, leptospirosis, Q fever, hepatitis E, West Nile virus (WNV), and Lyme disease. We detected *T. gondii* IgG and IgM by using VIDAS TOXO IgM and IgG II assays (bioMérieux, https://www.biomerieux.com). When *T. gondii* IgG was detected, we analyzed serum samples by using the Vidas Toxo IgG Avidity Assay (bioMérieux). Cutoff values used to interpret the results were those recommended by the manufacturers. All *Toxoplasma* spp. analyses were conducted at the LSPQ.

Other analyses were ordered. These analyses were detection of WNV IgM by ELISA using the WNV IgM Capture DxSelect (Focus Diagnostics, https://www.focusdx.com) at the LSPQ; detection of *Brucella* spp. IgM and IgG by using the standard tube *Brucella* agglutination test (in-house test at the LSPQ); detection of hepatitis E virus IgG and IgM by using a diagnostic assay (Wantai Biologic Pharmacy Enterprise, http://www.ystwt.cn); detection of *Leptospira* spp. IgM by using the Panbio *Leptospira* IgM ELISA (Abbott, https://www.abbott.com); detection of *Coxiella burnetii* IgG by using an immunofluorescence assay at the Centre Hospitalier Universitaire de Sherbrooke (Sherbrooke, Quebec, Canada); detection of *Borrelia burgdorferi* IgM and IgG by using the 2-tiered algorithm that included a screening ELISA conducted at Centre Hospitalier Universitaire de Sherbrooke (Zeus ELISA Borrelia VlsE1/pepC10 IgG/IgM test system; Alere, https://www.alere.com); an IgG Western blot assay (Anti-*Borrelia burgdorferi* U.S. EUROLINE-WB IgG; Euroimmun, https://www.euroimmun.com); and an IgM Line Blot Assay (Anti-*Borrelia* EUROLINE-RN-AT-adv IgM; Euroimmun) performed at the National Microbiology Laboratory in Winnipeg, Ontario, Canada.

## Results

### Epidemiologic Investigation

All 10 persons interviewed were men (age range 28–62 years). None of them had preexisting medical conditions. Clinical symptoms developed in 6 patients, including headache, fever, sweats, myalgia, and joint pain, during December 8–11, 2018; the earliest symptoms began a few days after the men returned home from Illinois. One case-patient was hospitalized because of severe headache, fever, and myalgia. Three other case-patients consulted a physician but were not hospitalized, and 2 other case-patients had similar symptoms but did not consult any physician.

We compiled results of *Toxoplasma* spp. testing and deer meat consumption for each hunter (Table). *Toxoplasma* spp. IgM was detected in 6 serum samples collected during the acute disease phase for the 6 symptomatic hunters. For 1 symptomatic hunter, *Toxoplasma* spp. IgM was detected when a second blood specimen was collected 3 weeks later. No IgG was detected in these serum samples, which suggested a recent infection in these hunters. We detected IgG with a low avidity index in the 6 serum samples collected from 6 case-patients during the convalescent phase, which enabled confirmation of a recent infection for all of these case-patients. For the 4 asymptomatic hunters, we analyzed 3 serum samples and detected IgG with a high avidity index in 2 samples, which suggested that both hunters were already immune to toxoplasmosis; 1 asymptomatic hunter was considered to be nonimmune. Even if this hunter consumed fresh deer meat that was undercooked, he did not show development of any disease and did not have any positive test results. One asymptomatic hunter did not participate in testing. When performed, serologic assays for hepatitis E, Q fever, leptospirosis, brucellosis, and Lyme disease all showed negative results.

**Table:** Characteristics of 10 hunters who had suspected toxoplasmosis, Quebec, Canada, 2018*

Hunter	Illness	Signs/symptoms	Consumption of deer meat	Collection date	Test	Result	Conclusion
1	Yes	Fever, sweats, cephalalgia, muscular, joint pain, fatigue	Rare steak	2018 Dec 16	IgM	Negative	No toxoplasmosis
					IgG II	Negative	
					IgG avidity	NA	
				2019 Jan11	IgM	Positive	Acute toxoplasmosis
					IgG II	Positive	
					IgG avidity	Low avidity	
2	Yes	Fever, sweats, cephalalgia, muscular, joint pain, fatigue	Rare steak	2018 Dec 21	IgM	Positive	Acute toxoplasmosis
					IgG II	Negative	
					IgG avidity	NA	
3	Yes	Fever, sweats, cephalalgia, muscular, joint pain, fatigue	Rare steak	2018 Dec 20	IgM	Positive	Acute toxoplasmosis
					IgG II	Equivocal	
					IgG avidity	NA	
				2019 Jan 14	IgM	Positive	Acute toxoplasmosis
					IgG II	Positive	
					IgG avidity	Low avidity	
4	Yes	Fever, cephalalgia, photophobic	Rare steak	2018 Dec 20	IgM	Positive	Acute toxoplasmosis
					IgG II	Negative	
					IgG avidity	NA	
				2019 Jan 14	IgM	Positive	Acute toxoplasmosis
					IgG II	Positive	
					IgG avidity	Low avidity	
5	Yes	Fever, sweats, cephalalgia, muscular, joint pain, fatigue	Medium steak	2018 Dec 19	IgM	Positive	Acute toxoplasmosis
					IgG II	Negative	
					IgG avidity	NA	
				2019 Jan 4	IgM	Positive	Acute toxoplasmosis
					IgG II	Positive	
					IgG avidity	Low avidity	
6	Yes	Fever, sweats, cephalalgia, muscular, joint pain, fatigue	Rare steak	2018 Dec 16	IgM	Positive	Acute toxoplasmosis
					IgG II	Negative	
					IgG avidity	NA	
				2019 Jan 4	IgM	Positive	Acute toxoplasmosis
					IgG II	Positive	
					IgG avidity	Low avidity	
7	No	Asymptomatic	Rare steak and heart	2019 Feb 7	IgM	Negative	Asymptomatic and nonimmune
					IgG II	Negative	
8	No	Asymptomatic	Well-done steak	2019 Jan 15	IgM	Negative	Asymptomatic: absence of IgM and high IgG avidity index excluded recent *Toxoplasma* spp. infection
					IgG II	Positive	
					IgG avidity	High avidity	
9	No	Asymptomatic	Well-done heart	2019 Jan 16	IgM	Negative	Asymptomatic: absence of IgM and high IgG avidity index excluded recent *Toxoplasma* spp. infection
					IgG II	Positive	
					IgG avidity	High avidity	
10	No	Asymptomatic	None	Did not participate	NA	NA	NA

*NA, not available.

We explored many possible risk factors to determine the most likely source of infection during the stay of the hunters, including water, food, and animal exposures. The hunters stayed at the camp for 12 days and during that time harvested and dressed 2–3 deer each. On November 30, the hunters prepared and consumed fresh deer steak that was cooked rare. Five of the 6 symptomatic hunters consumed rare steak, and 1 consumed steak cooked medium. Among the remaining 4 hunters who did not show development of symptoms, 1 consumed the uncooked heart of a deer, 2 consumed deer meat that was cooked well done, and 1 did not consume any deer meat on site. All other potential exposures were determined to be unremarkable. The Illinois Department of Public Health received reports of no similar illnesses in this area during the study period.

When the stay in Illinois ended, the hunters divided the remaining harvested meat among themselves and brought it back to Quebec. It was impossible to identify pieces of the deer partially consumed at the outfitter and possibly contaminated by *T. gondii.* At the beginning of the investigation, we recommended that the hunters not consume the deer meat until we knew more about the diagnosis. All deer meat was kept in freezers when the hunters returned home.

### Food Inspection Services by MAPAQ

We collected 12 samples of frozen deer meat from 1 hunter household: 7 specimens of 300 g each and 5 specimens of 500 g each from the same freezer. Among the 12 deer meat specimens collected for analysis, no *T. gondii* parasites were detected. Parts of deer that were consumed at the outfitter were unrecognizable from other parts of deer not consumed on site.

## Discussion

Outbreaks of acute toxoplasmosis infection are unusual in Quebec. In Illinois, no outbreaks were reported to the public health unit over a 20-year period. We identified a game meat–associated outbreak in Quebec involving travel to Illinois. Investigative findings identified consumption of fresh, undercooked deer meat as the most likely source of infection.

Sporadic cases associated with deer meat consumption have been reported (*13*). During 2017, acute toxoplasmosis developed in 8 of 10 hunters after they consumed fresh deer meat in Wisconsin, USA (*15*).

During the outbreak we report, symptoms were severe enough that 1 case-patient had to be hospitalized and 3 other companions consulted a physician. Primary acquired *Toxoplasma* spp. infection is predominantly asymptomatic in immunocompetent persons in North America (*17*). During the outbreak we report, 6 of 7 nonimmune hunters for whom we had the information showed development of symptoms after infection. The severity of infection might depend on the genotype of the strain. The severity of infection is usually low in North America, where genotype II strains predominate (*18*), in comparison to other parts of the world (*19**–**21*).

Another major outbreak of toxoplasmosis involving hundreds of persons was reported in 1995 in Victoria, British Columbia, Canada. The suspected source was an infected cougar that had defecated in the watershed; heavy rains had then washed a bolus of oocysts into the water reservoir. The outbreak included a high proportion of severe primary infections among immunocompetent persons (*18*). In the locations of that outbreak and the outbreak we report (British Columbia and Quebec), the genotype was not determined. We are not able to explain why so many cases were reported among the immunocompetent population.

In our investigation, we obtained serum samples from 3 of the 4 asymptomatic hunters. *Toxoplasma* IgG was detected in 2 serum samples. The absence of IgM and the high IgG avidity index suggest that both of those hunters were already immune to *T. gondii* by a past infection. One asymptomatic hunter did not show development of any disease and showed negative results for toxoplasmosis even after consuming fresh deer meat that was rare. We do not have immune information about the fourth patient.

Food specimens collected from 1 hunter were negative for *T. gondii*. Unfortunately, parts of the deer that were consumed at the outfitter were unrecognizable from parts of other deer harvested in Illinois during the same period of time, which might explain the negative results.

Little is known of the natural epidemiology of *T. gondii* infection in white-tailed deer. Given that deer are strict herbivores, it is believed that they become infected postnatally by ingesting oocysts from the environment (*7*). When ingested, the parasites form tissue cysts in the skeletal muscle and other tissues. When the infected deer die, tissues are scavenged by feline carnivore species, including bobcats and cougars (*7*). The life cycle then continues, and these cats shed more oocysts into the environment. Estimated *Toxoplasma* spp. prevalence among white-tailed deer varies across the United States from 15% to 74% (Ohio, Pennsylvania, and Minnesota) (*7**,**9**,**10**,**12*).

The MAPAQ website provides general recommendations to game meat hunters and their family about safe handling and preparation (*22*). Recommendations include not eating raw or undercooked game meat and cooking to an internal temperature of at least 160°F. They also recommend washing hands with soap and water after handling raw meat and cleaning all materials that come in contact with raw meat thoroughly after use. In addition, cysts and oocysts of toxoplasmosis might be destroyed by freezing the meat (*23**,**24*). Because the prevalence seems to be high in wild animals in which study prevalence was determined, freezing the meat seems to be efficient to destroy cysts and oocysts. Hunters should be aware of those recommendations. A person can be infected for life and disease can reactivate years after the initial infection.

Few studies have reported on the seroprevalence of *Toxoplasma* antibodies in humans in Canada. In the United States, a nationwide study conducted during 2009–2010 showed that the overall *T. gondii* antibody seroprevalence among persons >6 years of age was 13.2% (*24*). Other reports found that, although the presence of *T. gondii* is still relatively common, the prevalence in the United States decreased during 1988–1994 (*25*,*26*). Given the high prevalence of *Toxoplasma* spp. in white-tailed deer across some areas of the United States and the overall observed decrease in seroprevalence in humans, outbreaks like the one we reported might be more common in the future, and health professionals should be aware of this possibility.

In this investigation, we recommended to all hunters and their families that they not consume the deer meat. This recommendation was given even if all hunters were immunocompetent. If hunters and their families decided to consume the deer meat despite our recommendations, they were advised to freeze it thoroughly, cook it, and avoid distribution of the meat to family members, pregnant women, or immunocompromised persons.
